# Reply to the letter to the editor Transoral endoscopic thyroidectomy vestibular approach (TOETVA) and complications

**DOI:** 10.1590/0100-6991e-20213121

**Published:** 2021-08-13

**Authors:** GILBERTO MENDES MENDERICO

**Affiliations:** 1 - Centro Universitário Lusíada, Disciplina de Clínica Cirúrgica - Santos - SP - Brasil; 2 - Colégio Brasileiro de Cirurgiões - São Paulo - SP - Brasil

The article in the Brazilian College of Surgeons “Transoral Endoscopic Thyroidectomy Vestibular Approach (TOETVA) and complications”^1^ aimed to evaluate the potential complications of this relatively new thyroidectomy technique. We found data fulfilling the described method from the period between 2015 and the first week of 2020 (proved by the absence of articles from 2020), when the article was prepared and sent to the journal.

The authors performed the search for articles according to the rules, and flaws previously pointed out by the journal editors were corrected. The obtained data were compiled and then the article was written. Literature review is a “living” scientific method that can change over time, as new evidence is reached.

The article is the result of a scientific initiation project of medical students, guided by the main author, and was not intended to give personal opinions or make any value judgment regarding the surgical technique or those who perform it.

We would like to thank the authors of the letter to the editor for the contribution on the exposed literature findings, which strengthen the scientific discussion, and the editors of the Journal of the Brazilian College of Surgeons (CBC) for providing the opportunity of contradiction.

## References

[1] Bertelli AA, Lira RB, Gonçalves AJ, Kowalski LP (2021). Transoral endoscopic thyroidectomy vestibular approach (TOETVA) and complications.. Rev Col Bras Cir.

